# Evolution of health system resources and factors associated with the quality of care for epidemic-prone diseases in Guinea: a national cross-sectional study

**DOI:** 10.3389/fepid.2026.1818264

**Published:** 2026-08-28

**Authors:** Dimaï Ouo Kpamy, Fatoumata Cherif, Sory Conde, Fodé Amara Traore, Sidikiba Sidibe, Bassirou Diarra, Peter John Winch, Abdoulaye Toure, Sakoba Keita, Seydou Doumbia, Alexandre Delamou

**Affiliations:** 1 Faculty of Health Sciences and Technology, Gamal Abdel Nasser University, Conakry, Guinea; 2 Department of the Ministry of Health and Public Hygiene, National Agency for Health Security, Conakry, Guinea; 3 Department of Infectious Diseases, Infectious and Tropical Diseases Department of the Donka National Hospital, Conakry, Guinea; 4 Department of Public Health, African Center of Excellence for Prevention and Control of Communicable Diseases, Conakry, Guinea; 5 Department of Public Health, University of Sciences, Techniques and Technologies of Bamako (USTTB), Bamako, Mali; 6 Department of International Health, Johns Hopkins Bloomberg School of Public Health, Baltimore, MD, United States; 7 Department of Public Health, Research and Training Center in Infectiology, Conakry, Guinea

**Keywords:** associated factors, case management, epidemic-prone diseases, Guinea, management, quality, quality of care, resources

## Abstract

**Objective:**

This study aimed to describe the evolution of resources for the management of epidemic-prone diseases and to identify the factors associated with the quality of care for epidemic-prone diseases in Guinea.

**Methods:**

A descriptive and analytical cross-sectional study was conducted to assess the management system for epidemic-prone diseases in Guinea. Data were collected using structured questionnaires and supplemented by a review of official documents to assess the evolution of resources before, during, and after the 2014–2016 Ebola virus disease outbreak. Factors associated with the quality of care were identified using multivariable logistic regression analysis.

**Results:**

The evolution of resources varied across the different health regions. At the national level, the overall resource score increased from 12% before the Ebola outbreak to 73% during the outbreak and reached 77% in the post-Ebola period. Overall, the quality of care was rated as good in 60.5% of health facilities. Five factors were independently associated with good-quality care: the presence of a patient diagnostic mechanism (aOR = 3.17; 95% CI: 1.74–5.57; *p* < 0.001), the availability of treatment protocols (aOR = 2.42; 95% CI: 1.27–4.61; *p* = 0.007), the existence of patient isolation facilities (aOR = 4.76; 95% CI: 1.72–13.17; *p* = 0.003), effective coordination of case management (aOR = 3.04; 95% CI: 1.10–8.39; *p* = 0.031), and the availability of dedicated case management teams (aOR = 8.51; 95% CI: 4.07–17.76; *p* < 0.001).

**Conclusion:**

This study highlights a substantial improvement in the resources allocated to the management of epidemic-prone diseases in Guinea following the Ebola virus disease outbreak, reflecting strengthened health system capacity for epidemic preparedness and response. However, sustaining these gains will require continued investment, particularly in health system financing. Strengthening diagnostic capacity, ensuring the availability of standardized treatment protocols, expanding patient isolation facilities, improving coordination mechanisms, and maintaining specialized case management teams should remain strategic priorities to enhance the quality of care and strengthen the resilience of the health system against future epidemics.

## Introduction

Epidemic-prone diseases are infectious diseases that can affect multiple individuals simultaneously and spread from person to person within a defined geographic area (1).

The West African region has experienced several epidemics and outbreaks of infectious diseases, resulting in substantial morbidity and mortality in several countries and placing considerable strain on their health systems (2)⁠. These include anthrax in Guinea; cholera in Benin, Liberia, Nigeria, and Sierra Leone; dengue fever in Benin and Côte d'Ivoire; yellow fever in Nigeria; meningitis in Ghana, Niger, and Togo; and measles in Guinea, Niger, and Sierra Leone. In addition, Lassa fever is endemic in West Africa and has been reported in Sierra Leone, Guinea, Liberia, and Nigeria (3).

Guinea has experienced several public health emergencies, the most significant being the Ebola virus disease (EVD) outbreak between 2014 and 2016. Before this crisis, the Guinean health system was characterized by substantial weaknesses, including limited geographical coverage due to inadequate health infrastructure and equipment, overall system fragility, low access to healthcare services (38.9%), and low utilization of these services (18.6%) (4, 5). The Ebola outbreak exposed critical deficiencies in human resources, healthcare worker training, adequate health infrastructure, and access to healthcare services, particularly in rural areas (6). These structural weaknesses were further exacerbated during the epidemic by major challenges, including shortages of qualified healthcare workers, difficulties in the supply of essential medical commodities, limited coordination among response stakeholders, and delays in diagnosis and case management. In response, substantial measures were implemented to control the epidemic, including community mobilization strategies, the establishment of Community Care Centers (CCCs) and Ebola Treatment Centers (ETCs), capacity building for healthcare workers to improve early case detection, the systematic isolation of patients in ETCs, and the strengthening of infection prevention and control (IPC) measures in healthcare facilities (7, 8). Despite these efforts, several challenges persist, particularly those related to sustainable financing, logistics, community engagement, and the continuity of essential health services. During the post-Ebola recovery period, Guinea progressively strengthened its health system by establishing coordination, surveillance, diagnostic, and case management capacities at all levels of the health system. These initiatives included the establishment of the National Health Security Agency, the deployment of regional and prefectural epidemic alert and response teams, the creation of decentralized Public Health Emergency Operations Centers (PHEOCs), the development of hemorrhagic fever diagnostic laboratories, and the establishment of epidemic treatment centers (9–11). However, challenges remain, particularly with regard to the sustainability of financing, infrastructure maintenance, the continuous availability of essential medical commodities, and the equitable distribution of resources across regions. Currently, epidemic treatment centers are staffed by regularly trained healthcare personnel, ensuring the management of suspected or confirmed cases of epidemic-prone diseases and priority zoonotic diseases in accordance with established national protocols (6). The quality of care for epidemic-prone diseases depends on multiple interrelated factors (12–15). Although several studies have described the impact of the Ebola virus disease outbreak on health systems or evaluated the performance of epidemic response mechanisms in Guinea, they have primarily focused on assessing response capacities at a single point in time or during a specific epidemic. To our knowledge, no previous study has simultaneously examined the evolution of health system resources before, during, and after the Ebola outbreak while investigating the factors associated with the quality of care for epidemic-prone diseases in a context characterized by the occurrence of multiple epidemics. By combining a retrospective analysis of changes in human, material, financial, informational, and organizational resources with an analysis of the factors associated with the quality of care for epidemic-prone diseases, this study provides original evidence that enhances understanding of the long-term effects of post-Ebola investments on the capacity of the Guinean health system to respond to public health emergencies. These findings complement previous evaluations by identifying the health system components most strongly associated with high-quality care and highlighting the areas requiring further strengthening to improve the resilience of the health system to future epidemics.

### Research question

How have health system resources dedicated to the management of epidemic-prone diseases evolved in Guinea in the context of multiple epidemic outbreaks?

### Research hypothesis

The availability and organization of health system resources are associated with better quality of care for epidemic-prone diseases in Guinea.

## Study methods

### Study framework

**Figure 1:**
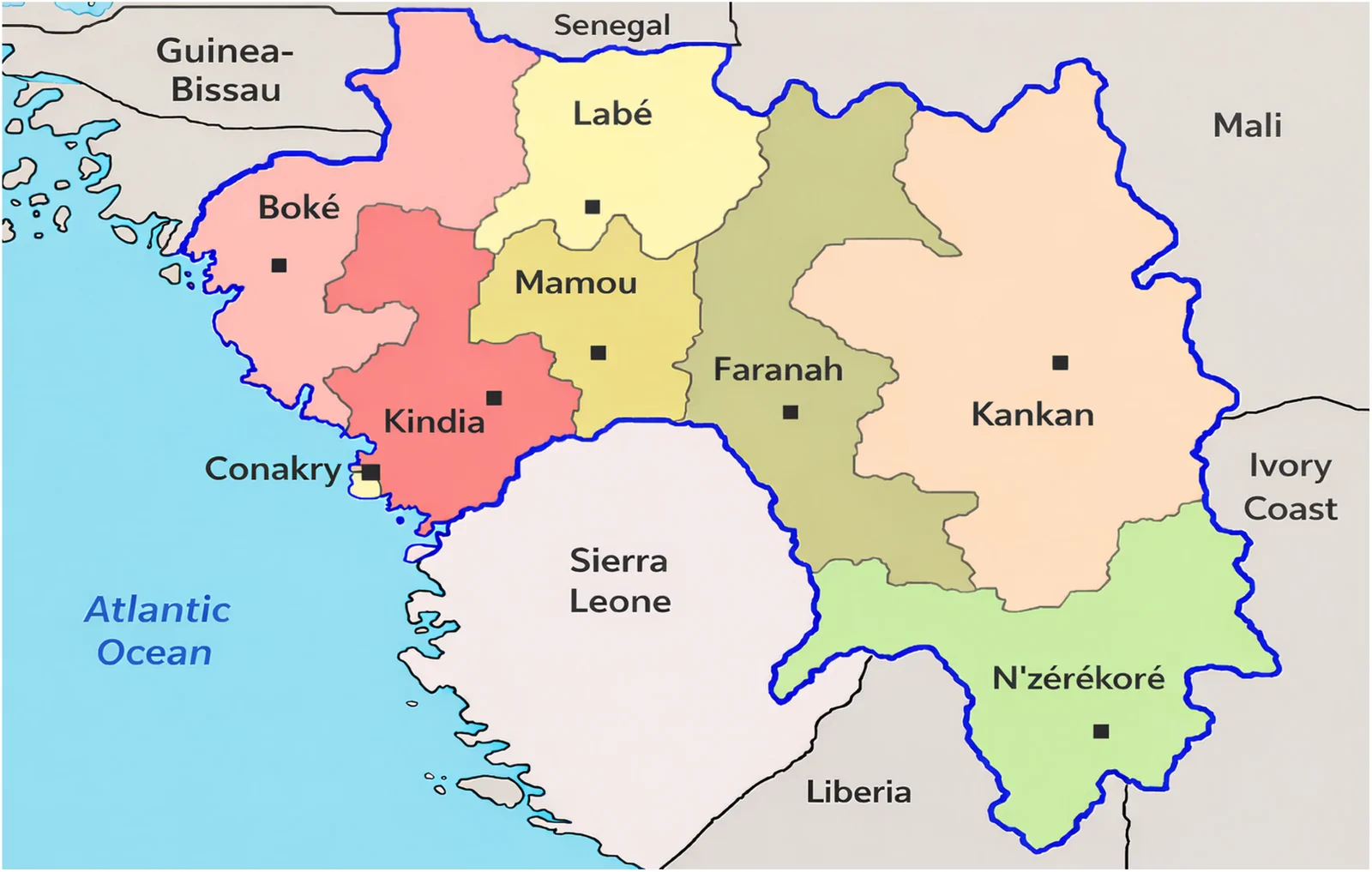
Health Map of Guinea showing the eight administrative regions (source: https://www.axl.cefan.ulaval.ca/afrique/guinee_fr-carteregionale.htm).

The Republic of Guinea is a coastal country located in West Africa, approximately midway between the Equator and the Tropic of Cancer, between latitudes 7°30′ and 12°30′ North and longitudes 8° and 15° West. It covers an area of 245,857 km^2^ and is bordered by Guinea-Bissau and the Atlantic Ocean to the west, Senegal and Mali to the north, Côte d'Ivoire to the east, and Sierra Leone and Liberia to the south (16–18). From a geoecological perspective, Guinea is divided into four distinct and internally homogeneous natural regions: Maritime Guinea, Middle Guinea, Upper Guinea, and Forest Guinea (18–21). Guinea is characterized by a diverse natural environment marked by climatic contrasts, mountainous landscapes, and varied topography, resulting in distinct regional specificities. Administratively, the country is divided into eight administrative regions, 33 prefectures, 38 urban municipalities including the five municipalities of Conakry and 303 rural municipalities (18, 19, 22, 23). Among the countries in West Africa, Guinea was the country most severely affected by the Ebola virus disease epidemic (24) (see Figure 1).

### Study site

The study was conducted in all three national directorates involved in epidemic response, as well as across Guinea's 38 health districts. It also included the country's eight Regional Health Inspectorates, 38 Prefectural Health Directorates, and 38 Epidemic Treatment Centers, all of which played key roles in epidemic preparedness and response in the Republic of Guinea.

### Study type

This was a nationwide descriptive cross-sectional study with an analytical component, conducted from April 2024 to October 2025. A mixed-methods approach was adopted to account for the diversity of the information sources.

### The target and source populations

The study population comprised all healthcare workers who were members of epidemic response committees in Guinea, including those from the technical departments of the Ministry of Health, Regional Health Inspectorates, Prefectural Health Directorates, and Epidemic Treatment Centers.

### Inclusion criteria

The study included actors who had participated in epidemic preparedness and response activities or in the coordination of epidemic response technical committees in Guinea.

### Exclusion criteria

Healthcare workers who had not participated in epidemic preparedness and response activities, as well as those who declined to participate or did not provide informed consent, were excluded from the study.

### Sampling

A probabilistic two-stage stratified sampling design was used, with administrative regions serving as the primary sampling units and epidemic response actors as the secondary sampling units. The selection of response actors within the health districts of the administrative regions was based on a comprehensive sampling frame developed with the support of operational teams deployed across the country's eight health regions. This sampling frame included, among others, Prefectural Health Directors, disease control physicians, planning, training and research officers, staff from Epidemic Treatment Centers, data managers, logisticians, laboratory managers, members of epidemic response committees, communication officers, hospital directors, personnel from the Public Health Emergency Operations Centers (PHEOCs), case management officers, and representatives of technical and financial partners.

Participants were then selected from this sampling frame within each health district of the administrative regions by systematic random sampling after verification of their eligibility according to the predefined inclusion and exclusion criteria. In cases of unavailability or refusal to participate, the next eligible individual on the sampling list was recruited to preserve the representativeness of the sample and achieve the planned sample size.

The minimum required sample size was calculated using the Schwartz formula.

$$n\;=\;Z\alpha^{2}\;(\;p\;\times \;q)/i^{2}$$

with:

Z*α* is the standard normal deviation for a 95% confidence level and is equal to 1.96
*p* = expected proportion 50% = 0.5
*q* = 1 − *p* = 0.5
*d* = desired margin of error = 5% = 0.05
*n* = minimum sample size

Overall, 400 epidemic response actors were included in the study out of the 428 initially planned, corresponding to a participation rate of 93.5%.

### Study variables

#### Identical variables related to the evolution of resources before, during, and after the Ebola virus disease (EVD) outbreak

1. Human resources: availability of trained healthcare workers, availability of sufficient human resources for case management, existence of a supervision plan for case management personnel, and existence of a coordination mechanism for case management activities; 2. material resources: availability of functional isolation facilities, availability of medicines and medical supplies for case management, availability of diagnostic laboratories, availability of functional mobile logistics for case management, availability of a computer at the case management site, and availability of a printer at the case management site; 3. financial resources: availability of funding for case management and provision of food for patients receiving care; 4. information resources: availability of case management guidelines, availability of case definition forms, existence of a case notification system, availability of a patient referral protocol, and existence of mechanisms to ensure continuity of care for patients with complications; 5. time resources: prompt case management.

#### Dependent variable

The dependent variable was the quality of care for epidemic-prone diseases. It was a dichotomous variable and was assessed based on the presence or absence of appropriate case management practices for these diseases. The criteria used to assess the quality of care for epidemic-prone diseases were as follows (25–27): The dependent variable was the quality of care for epidemic-prone diseases. It was a dichotomous variable and was assessed based on the presence or absence of appropriate case management practices for these diseases. The criteria used to assess the quality of care for epidemic-prone diseases were as follows: The quality of care was assessed using the following criteria: (1) early diagnosis of patients; (2) timeliness of patient care; (3) early patient isolation; (4) regular organization of care coordination meetings; (5) availability of dedicated case management staff; (6) safety of patient care; and (7) administration of safe treatments in accordance with established protocols. This composite score does not correspond to an internationally validated scale but was developed using recognized quality-of-care indicators adapted to the Guinean context. Before its implementation, the tool was pretested to assess its clarity, relevance, and feasibility.

The quality of care was classified as follows:

◦ Good-quality care (score = 1): fulfillment of at least six (6) quality criteria, including the administration of safe treatments in accordance with established protocols;
◦ Poor-quality care (score = 0): fulfillment of fewer than six (6) quality criteria, including the absence of safe treatment administration in accordance with established protocols.

#### Independent variables

Presence of a functional isolation facility, availability of healthcare personnel trained in the management of epidemic-prone diseases (EPDs), availability of medicines and medical supplies, presence of a functional laboratory for EPD diagnosis, availability of diagnostic tests, availability of functional mobile logistics, availability of dedicated funding for case management during epidemics, availability of an EPD case management guideline, presence of a functional water supply (borehole) at the isolation facility, support from technical and financial partners, availability of infection prevention and control (IPC) kits at the isolation facility, availability of a healthcare waste management area at the isolation facility, availability of a case management protocol, existence of a psychosocial care mechanism, availability of an infection prevention and control (IPC) manual, existence of an EPD diagnostic protocol, availability of a continuity of care plan for severe or complicated cases, existence of a supervision plan for healthcare providers, availability of a medicines and medical supplies procurement plan, existence of an isolation facility rehabilitation plan, availability of a patient referral protocol, existence of a case notification mechanism, and the presence of a coordination mechanism for EPD case management activities during outbreaks.

### Data collection techniques and tools

The data were collected primarily through individual interviews. To analyze the evolution of resources before, during, and after the Ebola epidemic, the self-reported data obtained from these interviews were supplemented and verified using official documentary sources, including national preparedness and response plans, epidemiological surveillance reports, supervision reports, investigation reports, training reports, national clinical management guidelines and protocols, epidemic treatment center registers, as well as administrative documents from the Ministry of Health and the National Health Security Agency (ANSS). This triangulation made it possible to reconstruct the evolution of human, material, financial, informational, and organizational resources across the different periods under study.

### Data validity

To minimize the recall bias inherent in this retrospective approach, several measures were implemented: (i) the selection of respondents who were directly involved in epidemic management during the study periods; (ii) the use of a standardized questionnaire based on predefined indicators; (iii) the systematic cross-validation of the information collected against available administrative and technical documents (data triangulation); and (iv) quality control procedures were implemented at every stage of the data management process including data collection, entry, cleaning, and analysis to minimize processing errors and ensure the consistency of the data used for statistical analyses. This approach strengthened the validity of the collected information and reduced the risk of recall bias in the assessment of changes in health system resources.

### Data processing and analysis

Data were entered and managed using KoboToolbox. Following data cleaning, only complete and analyzable questionnaires were retained for statistical analyses. No missing data were identified for the variables included in the analyses; therefore, no imputation method was applied. All statistical analyses were performed using Stata version 13. Descriptive analyses were conducted to summarize the characteristics of the study population and the distribution of the study variables. Qualitative variables were described using absolute frequencies and percentages, whereas quantitative variables were summarized as means and standard deviations.

National aggregated data were used exclusively to describe the evolution of health system resources. Analytical analyses, including bivariate and multivariable logistic regression, were performed using individual-level data.

The analysis of the evolution of resources for the management of epidemic-prone diseases focused on changes in health system resources before, during, and after the Ebola virus disease outbreak in Guinea. This analysis was conducted in two stages. The first stage examined changes in resources at the regional level across the country's eight administrative regions, while the second provided an overall assessment at the national level. Changes in health system resources were interpreted using the World Health Organization (WHO) criteria for the classification of health system resources (28–30). The national evolution of resources dedicated to the management of epidemic-prone diseases was estimated from the regional averages calculated across the eight administrative regions. For each resource category, a regional percentage was derived from the indicators observed at the health district level, and the mean of these regional percentages was then calculated to estimate the national level. Finally, the overall national evolution of resources was determined by averaging the percentages across the different resource categories. The levels of resource evolution were subsequently assessed using the measurement scale proposed by Varkevisser (31). This evaluation allowed the results to be classified into the following categories (31):

- “Good evolution”: for a percentage between 80% and 100%;
- “Average evolution”: for a percentage between 60% and 80%;
- “Poor evolution”: for a percentage between [0% and 60%].

The quality of care was considered good when at least six criteria were met, with adherence to national treatment protocols through the administration of protocol-compliant treatments as an essential requirement. This threshold was defined using a normative approach, based on the principle that a high level of compliance with good clinical practices is necessary to ensure high-quality care in the context of epidemic-prone diseases. Factors associated with the quality of care for epidemic-prone diseases were identified using individual-level data collected in 2025. The analyses were conducted using a multistep approach:

- First, a univariable analysis was performed to assess the association between the dependent variable and each independent variable using simple binary logistic regression. Crude odds ratios (ORs), their 95% confidence intervals (95% CIs), and *p* values were estimated.
- Variables with a *p* value <0.20 in the univariable analysis, together with those considered epidemiologically relevant, were entered into an initial multiple logistic regression model. A backward stepwise logistic regression procedure was then applied to sequentially eliminate the least contributive variables until the final model was obtained. Associations were expressed as adjusted odds ratios (aORs) with their corresponding 95% confidence intervals. Variables with a *p* value <0.05 were considered statistically associated with the quality of care and were retained in the final model.
- Before interpreting the final model, multicollinearity among the explanatory variables was assessed using the Variance Inflation Factor (VIF). VIF values were interpreted according to commonly accepted thresholds in the literature: a VIF of 1 indicates the absence of collinearity; values between 1 and 2 indicate very low collinearity and are generally considered excellent; values between 2 and 5 indicate low collinearity and are considered acceptable; values between 5 and 10 suggest moderate collinearity requiring particular attention; and a VIF greater than 10 is considered indicative of high collinearity that may compromise the stability and interpretation of the regression model coefficients.
- Finally, the goodness of fit of the final model was evaluated using the Hosmer–Lemeshow test. The model was considered to have an adequate fit when the *p* value was >0.05, indicating satisfactory agreement between the predicted probabilities and the observed outcomes.

### Ethical considerations

Ethical approval for the study was obtained from the National Ethics Committee for Health Research (CNERS) (Approval No. 122/CNERS/24), as well as authorization to collect data from the General Directorate of the National Health Security Agency. All participants received an information sheet, and written informed consent was obtained before the administration of the structured questionnaire and the conduct of individual interviews. Participant anonymity and data confidentiality were strictly maintained throughout the data collection, management, and analysis processes.

## Results

### Sociodemographic and professional characteristics of the study participants

A total of 400 epidemic response actors from the eight administrative regions of Guinea were included in the study. Of the participants, 75% were men and 25% were women, corresponding to a male-to-female ratio of 3:1. The mean age was 52.33 ± 6.23 years. Overall, 43% of participants were younger than 40 years, 54% were between 40 and 60 years of age, and 3% were older than 60 years. Regarding educational attainment, the vast majority of participants (91.77%) held a university degree, whereas 8.23% had completed secondary education.

### Overall evolution of health system resources for the management of epidemic-prone diseases at the national level in Guinea before, during, and after the Ebola virus disease outbreak

At the national level, health system resources dedicated to the management of epidemic-prone diseases improved substantially between the pre-Ebola, Ebola, and post-Ebola periods. Human, material, informational, and temporal resources increased markedly during the Ebola outbreak, and these gains were largely sustained thereafter. Human resources increased from 12% before Ebola to 74% during the outbreak and 79% afterward. Similarly, material resources rose from 8% to 72% during the outbreak and reached 78% in the post-Ebola period. Informational resources showed continuous improvement, increasing from 18% before Ebola to 79% during the outbreak and 89% afterward, while temporal resources increased from 15% to 67% and then to 87%, reflecting sustained improvements in surveillance systems, health information management, and the timeliness of response. Financial resources also improved substantially during the outbreak, increasing from 9% to 71%, before declining to 50% in the post-Ebola period, suggesting challenges in sustaining funding once the emergency phase had ended. Overall, the composite resource score increased from 12% before the outbreak to 73% during the Ebola period and 77% afterward, indicating a transition from a poor level of resource availability before the outbreak to an average level that was maintained during and after the Ebola outbreak (see Table 1).

**Table 1:** Overall evolution of resources for the management of epidemic-prone diseases at the national level in Guinea before, during, and after the Ebola virus disease outbreak, 2025.

National level resources	Before EVD	During EVD	After EVD
	*N* = 400	*N* = 400	*N* = 400
Human resources	47 (12%)	299 (74%)	316 (79%)
Material resources	34 (8%)	290 (72%)	315 (78%)
Financial resources	37 (9%)	286 (71%)	60 (15%)
Informational resources	69 (18%)	317 (79%)	355 (89%)
Temporal resources	62 (15%)	267 (67%)	348 (87%)
Overall evolution of resources	**49.8** **(12%)**	**291.8** **(73%)**	**307** **(77%)**
Assessment of the evolution	Poor	Average	Average

EVD: Ebola virus disease.

Bold values indicate the overall summary values of the individual resource categories.

### Factors associated with the quality of care for epidemic-prone diseases in Guinea

**Figure 2:**
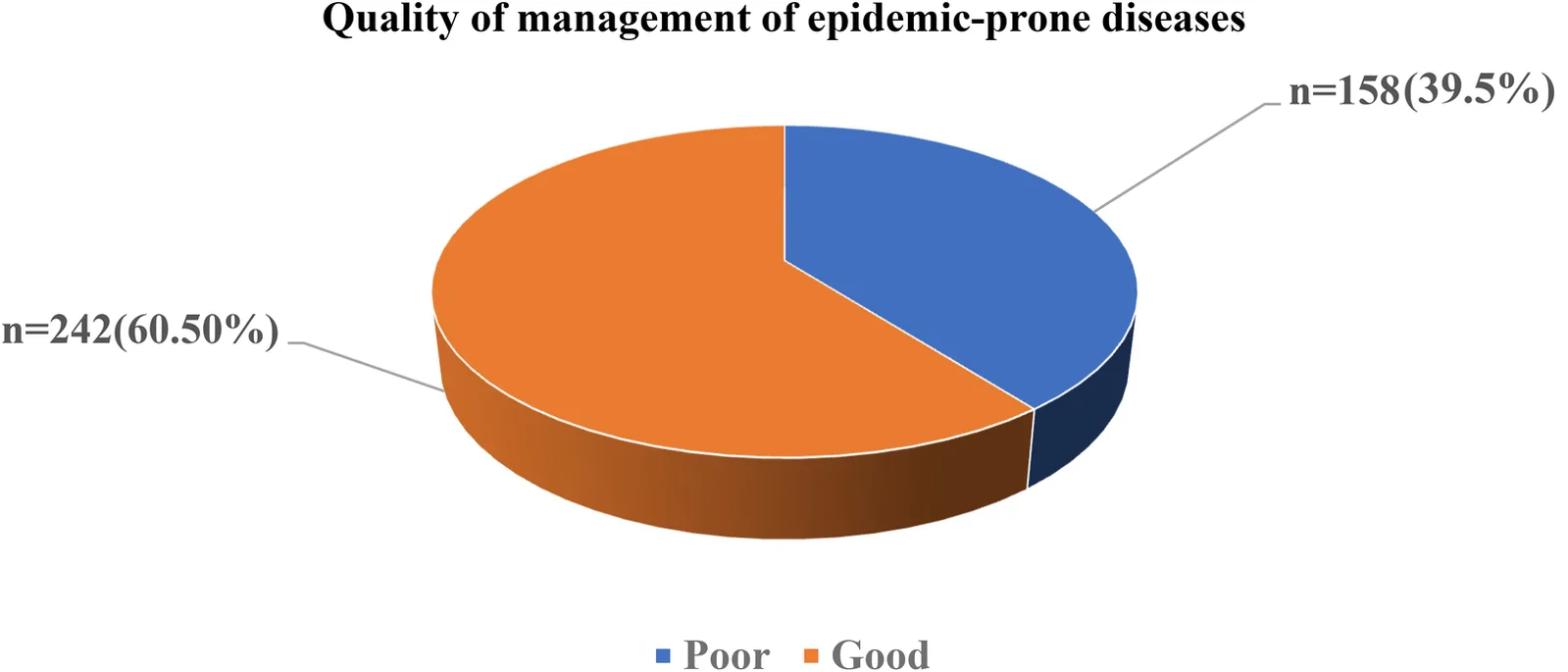
Quality of management of epidemic-prone diseases in Guinea, 2025.

Overall, the quality of care for epidemic-prone diseases in Guinea was predominantly rated as good, accounting for 60.5% of cases, whereas 39.5% were classified as having poor quality of care (see Figure 2).

### Factors associated with the quality of care for epidemic-prone diseases in Guinea according to univariate logistic regression analysis

Univariable logistic regression analysis identified several factors significantly associated with the quality of care for epidemic-prone diseases. The presence of a patient diagnostic mechanism was associated with nearly a fourfold higher odd of good-quality care compared with its absence (OR = 3.91; 95% CI: 2.39–6.40; *p* < 0.001). Likewise, the availability of case management protocols (OR = 5.76; 95% CI: 2.54–13.09; *p* < 0.001), the existence of functional isolation facilities (OR = 7.75; 95% CI: 3.11–19.30; *p* < 0.001), healthcare waste management (OR = 2.40; 95% CI: 1.57–3.88; *p* < 0.001), continuity of care (OR = 2.80; 95% CI: 1.17–4.40; *p* < 0.001), water supply (OR = 2.80; 95% CI: 1.17–4.40; *p* < 0.001), the availability of medicines and medical supplies (OR = 3.40; 95% CI: 2.11–5.50; *p* < 0.001), support from technical and financial partners (OR = 3.10; 95% CI: 1.93–4.96; *p* < 0.001), effective coordination of case management (OR = 9.63; 95% CI: 3.25–28.51; *p* < 0.001), and the availability of dedicated case management teams (OR = 9.44; 95% CI: 4.72–18.87; *p* < 0.001) were all significantly associated with higher odds of good-quality care.

In contrast, the availability of a psychosocial care mechanism was associated with significantly lower odds of good-quality care (OR = 0.33; 95% CI: 0.18–0.60; *p* = 0.001). However, the availability of dedicated funding for case management was not significantly associated with the quality of care (OR = 1.06; 95% CI: 0.60–1.86; *p* = 0.841) (see Table 2).

**Table 2:** Factors associated with the quality of management of epidemic-prone diseases in Guinea according to univariate logistic regression analysis, 2025.

Factors associated	Frequency (*n*)	OR	95% CI	*P* value
Presence of a patient diagnostic mechanism				
No	91	1	—	—
Yes	309	3.91	[2.39–6.40]	0.000
Availability of treatment protocols for patients				
No	34	1	—	—
Yes	366	5.76	[2.54–13.09]	0.000
Existence of isolation sites for patients				
No	32	1	—	—
Yes	368	7.75	[3.11–19.30]	0.000
Healthcare waste management				
No	108	1	—	—
Yes	292	2.4	[1.57–3.88]	0.000
Continuity of care				
No	103	1	—	—
Yes	297	2.8	[1.17–4.4]	0.000
Water supply				
No	109	1	—	—
Yes	291	2.8	[1.17–4.4]	0.000
Availability of medicines and medical supplies				
No	95	1	—	—
Yes	305	3.4	[2.11–5.50]	0.000
Support from technical and financial partners				
No	98	1	—	—
Yes	302	3.10	[1.93–4.96]	0.000
Coordination of care				
No	29	1	—	—
Yes	371	9.63	[3.25–28.51]	0.000
Availability of care teams				
No	50	1	—	—
Yes	350	9.44	[4.72–18.87]	0.000
Psychosocial care for patients				
No	346	1	—	—
Yes	54	0.33	[0.18–0.60]	0,001
Funding for care				
No	340	1	—	—
Yes	60	1.06	[0.6–1.86]	0,841

OR, odds ratio.

### Factors associated with the quality of care for epidemic-prone diseases in Guinea according to multivariable logistic regression analysis

Multivariable logistic regression analysis identified five factors independently associated with the quality of care for epidemic-prone diseases. After adjustment for the other variables included in the model, the presence of a patient diagnostic mechanism remained associated with a more than threefold higher likelihood of providing good-quality care compared with its absence (aOR = 3.17; 95% CI: 1.74–5.57; *p* < 0.001). Similarly, the availability of patient management protocols was significantly associated with improved quality of care (aOR = 2.42; 95% CI: 1.27–4.61; *p* = 0.007). The existence of isolation facilities (aOR = 4.76; 95% CI: 1.72–13.17; *p* = 0.003) and effective coordination of patient care (aOR = 3.04; 95% CI: 1.10–8.39; *p* = 0.031) also remained independently associated with good-quality care for epidemic-prone diseases. Furthermore, the availability of case management teams was the factor most strongly associated with the quality of care, with facilities having such teams showing more than an eightfold higher likelihood of providing good-quality care than those without them (aOR = 8.51; 95% CI: 4.07–17.76; *p* < 0.001). Finally, the assessment of multicollinearity showed variance inflation factor (VIF) values ranging from 1.08 to 1.30, with a mean VIF of 1.18, indicating very low collinearity among the explanatory variables and confirming the absence of multicollinearity likely to affect the stability of the model estimates (See Table 3).

**Table 3:** Factors associated with the quality of care for epidemic-prone diseases in Guinea according to multivariable logistic regression analysis, 2025.

Factors associated	Frequency (*n*)	aOR	95% CI	*P* value	Multicollinearity (VIF) Mean—VIF = 1.18
Presence of a patient diagnostic mechanism					
No	91	1	—	—	–
Yes	309	3.17	[1,74–5,57]	0,000	1.30
Availability of treatment protocols for patients					
No	34	1	—	—	–
Yes	366	2.42	[1.27–4.61]	0,007	1.30
Existence of isolation sites for patients					
No	32	1	—	—	–
Yes	368	4.76	[1.72–13.17]	0,003	1.11
Coordination of care					
No	29	1	—	—	—
Yes	371	3.04	[1.10–8.39]	0,031	1.09
Availability of care teams					
No	60	1	—	—	—
Yes	340	8.51	[4.07–17.76]	0,000	1.08

aOR, adjusted odds ratio.

## Discussion

### Evolution of resources for the management of epidemic-prone diseases

This study highlights a substantial improvement in health system resources devoted to the management of epidemic-prone diseases in Guinea across the pre-Ebola, Ebola, and post-Ebola periods. The progress observed primarily concerned human, material, informational, and temporal resources, reflecting the gradual strengthening of national capacities for preparedness, detection, and response to public health emergencies following the Ebola virus disease outbreak. In contrast, the evolution of financial resources appeared less sustainable, with a decline observed during the post-Ebola period, although their level remained higher than during the pre-epidemic period.

The improvement in human resources observed in this study is consistent with the major reforms undertaken following the Ebola outbreak. The 2014–2016 crisis highlighted shortages of qualified personnel for the surveillance, prevention, and management of infectious diseases. In response, the Guinean health authorities, with the support of technical and financial partners, invested in the recruitment of health professionals, continuing education, and the strengthening of competencies in public health emergency management. These investments notably enabled the deployment of training programs in field epidemiology through the Field Epidemiology Training Program (FETP), infection prevention and control, hospital hygiene and patient safety, case management, and public health emergency management (32, 33). They also resulted in the establishment and strengthening of specialized response teams, including the Regional Epidemic Alert and Response Teams (ERARE), the Prefectural and Municipal Epidemic Alert and Response Teams (EPARE/ECARE), and the national Strengthening and Utilizing Response Groups for Emergencies (SURGE) teams. Similar observations have been reported in several West African countries, where the Ebola outbreak served as a powerful catalyst for strengthening human resources and the institutional capacities of health systems (34–36).

The substantial increase in material resources reflects investments in health infrastructure, laboratories, personal protective equipment, epidemic treatment centers (CTEPI), and logistical systems. These findings are consistent with recommendations arising from international assessments of the Ebola response, which emphasized the need to sustainably strengthen the operational capacities of high-risk countries. The maintenance of a high level of material resources after the outbreak indicates improved health system preparedness for emerging and re-emerging health threats such as COVID-19, Marburg virus disease, mpox, and Lassa fever (37).

Informational resources showed the greatest improvement during the study period. This evolution reflects the strengthening of the national integrated disease surveillance and response system, the deployment of electronic data management platforms, particularly DHIS2 and the SAP database, the standardization of case management protocols and guidelines, and the improvement of mechanisms for coordinating and sharing health information (38–40). These advances are consistent with the requirements of the International Health Regulations (2005) and the recommendations of the Joint External Evaluation (JEE), which recognize health information systems as a core health security capacity (41).

Temporal resources also improved considerably through the adoption of the 7-1-7 approach, which aims to detect a public health event within a maximum of seven days, notify it within 24 h of detection, and implement the main response actions within the following seven days (42–45). This evolution resulted in shorter delays in detection, notification, investigation, and case management. It may be attributed to the strengthening of epidemic treatment centers (CTEPI), the operationalization of Public Health Emergency Operations Centers (PHEOC), the development of the diagnostic capacities of national and regional laboratories, and the improvement of multisectoral coordination. Reducing response time is a major determinant of outbreak control, as it limits pathogen transmission and improves the effectiveness of public health interventions.

In contrast, although financial resources increased substantially during the Ebola outbreak, their decline after the crisis highlights the difficulties involved in ensuring sustainable financing for public health emergency preparedness and response activities. This trend is frequently observed in low- and middle-income countries, where international funding increases considerably during crises before declining rapidly once outbreaks have been brought under control. Such dependence on external funding constitutes a major vulnerability for health system resilience and compromises the sustainability of gains achieved during emergency situations.

Overall, the improvement in resources observed in this study reflects the positive effects of the investments made following the Ebola outbreak. However, persistent disparities among the different categories of resources, particularly financial resources, show that progress remains partly dependent on support from technical and financial partners. The sustainable consolidation of these gains will require stronger government commitment to sustainable domestic financing, long-term strategic planning, and the continuous strengthening of national capacities for preparedness, early detection, and response to public health emergencies.

Ultimately, these findings confirm that the Ebola outbreak marked a major turning point in the strengthening of Guinea's health system. Although substantial progress has been achieved in most of the components assessed, the sustainability of these gains will depend on the country's capacity to maintain investments beyond periods of crisis, reduce its dependence on external funding, and consolidate a resilient health system capable of effectively preventing, detecting, and responding to future epidemic threats.

### Factors associated with the quality of care for epidemic-prone diseases in Guinea

This study identified five factors independently associated with the quality of care for epidemic-prone diseases in Guinea: the existence of a patient diagnostic mechanism, the availability of treatment protocols, the existence of patient isolation facilities, effective coordination of case management, and the availability of dedicated case management teams (34, 46). These findings indicate that the quality of care for epidemic-prone diseases depends not only on clinical expertise but also on the organization of health services and the availability of essential structural and operational resources (27, 34).

The presence of a patient diagnostic mechanism was independently associated with better quality of care. Early and accurate diagnosis is the cornerstone of effective epidemic management, as it enables the rapid identification of cases, appropriate clinical management, the early implementation of infection prevention and control measures, and timely notification to surveillance systems. During infectious disease outbreaks, delayed or inaccurate diagnosis facilitates ongoing transmission, increases morbidity, and contributes to higher mortality. Similar findings were reported during the Ebola virus disease outbreak in West Africa, where strengthened diagnostic capacity, particularly through the expansion of laboratory services, the use of standardized case definitions, and the adoption of rapid diagnostic algorithms, substantially improved both clinical outcomes and outbreak control. Comparable results were also observed during the COVID-19 pandemic, when countries with stronger diagnostic capacities achieved earlier case detection and more effective patient management (35, 47, 48).

The availability of treatment protocols was also significantly associated with better quality of care. Standardized clinical protocols reduce variations in medical practice, facilitate evidence-based clinical decision-making, reinforce adherence to national and international guidelines, and promote consistent patient management across health facilities. During public health emergencies, the regular updating of these protocols is particularly important because recommendations evolve rapidly as new scientific evidence becomes available. Similar findings have been reported in studies of Ebola, COVID-19, and other emerging infectious diseases, demonstrating that standardized protocols improve both the quality and consistency of care while reducing preventable complications (35).

The existence of patient isolation facilities was another important factor associated of the quality of care. Isolation remains one of the most effective measures for interrupting disease transmission within healthcare facilities while ensuring appropriate clinical management of infected patients (49). During outbreaks of Ebola virus disease, COVID-19, mpox, and other highly transmissible infections, specialized isolation units have proven effective in reducing healthcare-associated infections, protecting healthcare workers, and improving clinical outcomes through specialized patient care. In Guinea, the establishment of treatment centers and isolation facilities following the Ebola outbreak substantially strengthened national capacities for epidemic preparedness and response (35).

Effective coordination of case management was also independently associated with better quality of care. Epidemic response requires continuous coordination among clinicians, surveillance teams, laboratories, logistics units, infection prevention and control specialists, risk communication teams, and decision-makers (50). Well-coordinated health systems facilitate the rapid mobilization of resources, improve information sharing, strengthen multidisciplinary collaboration, and ensure continuity of patient care. These findings are consistent with those reported by the World Health Organization and several studies conducted during the Ebola and COVID-19 responses, which identified coordination as a major determinant of response effectiveness and health system resilience (51–54).

The strongest association observed in this study was related to the availability of dedicated case management teams. Health facilities with specialized multidisciplinary teams were more than eight times more likely to provide good-quality care than those without such teams. These specialized teams contribute to improved clinical decision-making, effective implementation of infection prevention and control measures, rapid application of treatment protocols, and efficient management of severe cases. Experience gained during the Ebola outbreak in Guinea demonstrated that specialized case management teams, supported by continuous training and simulation exercises, substantially improved clinical performance and patient safety (55). Similar observations were also reported during the COVID-19 pandemic, where multidisciplinary response teams enhanced both clinical outcomes and operational effectiveness (56, 57).

The absence of problematic multicollinearity confirms that each of these factors independently contributed to explaining the observed variation in the quality of care. Overall, these findings suggest that improving the quality of care for epidemic-prone diseases requires sustained investments in diagnostic capacity, the development and regular updating of standardized clinical protocols, the strengthening of isolation infrastructure, the improvement of multidisciplinary coordination mechanisms, and the development of specialized response teams. Strengthening these components should constitute a strategic priority for enhancing the preparedness and resilience of the health system in the face of future public health emergencies.

### Implications for future epidemic preparedness and response

The findings of this study have several important implications for strengthening preparedness and response to future epidemics in Guinea. Although the quality of care for epidemic-prone diseases was generally considered satisfactory, sustaining this level of performance will depend on the health system's capacity to preserve and consolidate the gains achieved since the 2014–2016 Ebola virus disease outbreak.

The five factors independently associated with good-quality care the availability of a patient diagnostic mechanism, the existence of standardized treatment protocols, the availability of functional isolation facilities, effective coordination of case management, and the availability of dedicated case management teams should constitute the cornerstones of future national epidemic preparedness strategies. Strengthening these components will not only improve the quality of care during epidemic outbreaks but also reduce delays in case detection, limit the transmission of infectious agents, and optimize the use of available resources.

The findings also underscore the need to sustain long-term investments in a qualified health workforce, health infrastructure, diagnostic capacity, laboratory systems, disease surveillance systems, and multisectoral coordination mechanisms. In this regard, the decline in financial resources observed during the post-Ebola period represents a major threat to the sustainability of the capacities developed. Without predictable, flexible, and sustainable financing mechanisms, the progress achieved in epidemic preparedness and response may gradually erode, thereby compromising the country's ability to respond effectively to future public health emergencies (34, 35, 58). In this context, national policies should give particular priority to strengthening rapid response teams, maintaining diagnostic capacity, regularly updating evidence-based case management protocols, ensuring that isolation facilities remain fully operational, and enhancing coordination across all levels of the health system and among sectors involved in the One Health approach. These investments will contribute to strengthening the long-term resilience of Guinea's health system, improving the quality of care during future epidemics, and consolidating national and regional health security in accordance with the recommendations of the International Health Regulations (2005) and the Global Health Security framework (59–62).

### Study limitations

This study has several limitations that should be considered when interpreting the findings:

- First, due to operational constraints related to the implementation of field activities, some participants initially targeted could not be interviewed or complete the questionnaires, which may have slightly affected the participation rate.
- Second, the cross-sectional design of the study does not allow causal relationships to be established between the identified factors and the quality of care.
- Third, the retrospective collection of information on the evolution of health system resources may have introduced recall bias. However, this potential bias was minimized through the selection of respondents who were directly involved in epidemic response activities, the use of a standardized questionnaire, and the systematic triangulation of the collected data with official documents.

Despite these limitations, the national coverage of the study, the triangulation of multiple data sources, and the use of a multivariable analytical approach strengthen the robustness and credibility of the findings.

## Conclusion

This study demonstrated that the quality of care for epidemic-prone diseases in Guinea is generally satisfactory, reflecting the progress achieved in strengthening national preparedness and response capacities since the 2014–2016 Ebola virus disease outbreak. It also highlights substantial improvements in the human, material, organizational, and technical resources of the health system during the post-Ebola period, although a decline in financial resources was observed.

The study identified five factors independently associated with good-quality care: the presence of a patient diagnostic mechanism, the availability of standardized case management protocols, the existence of functional isolation facilities, effective coordination of care, and the availability of dedicated case management teams. These findings underscore that improving the quality of care during epidemics depends on a combination of technical capacity, efficient health service organization, and the availability of adequate resources.

To preserve the gains achieved and strengthen the resilience of the health system in the face of future public health emergencies, sustained investments in human resources, diagnostic capacity, health infrastructure, coordination mechanisms, and sustainable financing remain essential. These priorities will contribute to the long-term improvement of the quality of care for epidemic-prone diseases and strengthen health security in Guinea.

## Data Availability

The original contributions presented in the study are included in the article/Supplementary Material, further inquiries can be directed to the corresponding author.
